# The ameliorating effect of the combined extract from Greek *Thymus vulgaris* and bee's honey on the hydrocortisone-induced osteoporosis in rat bone cells *via* modulating the bone turnover, oxidative stress, and inflammation

**DOI:** 10.1039/c8ra04370a

**Published:** 2018-08-07

**Authors:** Marwa M. Abu-Serie, Noha H. Habashy

**Affiliations:** Department of Medical Biotechnology, Genetic Engineering, Biotechnology Research Institute, City for Scientific Research and Technology Applications (SRTA-City) New Borg EL-Arab 21934 Alexandria Egypt; Biochemistry Department, Faculty of Science, Alexandria University Alexandria 21511 Egypt noha.Habashi@alexu.edu.eg nohahhm@gmail.com +20-2(03) 3911794 +20 1273431731

## Abstract

Many of the functional foods are designed to decrease the risk of chronic diseases like osteoporosis (OP) which is the most common bone disorder affecting millions of people. For the first time, the present study evaluated the effect of the combination between the Greek *Thymus vulgaris* water extract (TVE) and bee's honey (BH) against hydrocortisone (HC)-induced OP *in vitro*. The characterization of TVE, BH, and their combined extract (TV–BH) was examined. In addition, the current work assessed the bone turnover, oxidative stress, and inflammatory markers in bone cells. The results revealed the presence of considerable amounts of phenolics, flavonoids, anthocyanins, and flavonols in TVE and BH as well as important minerals and vitamins for the bone health. The TV–BH showed a synergistic (combination index <1) attenuation effect for the HC-induced bone cell damage through significant (*p* < 0.05) up-regulation of the hydroxyapatite, osteocalcin, phosphorous, and collagen contents. In addition, it significantly (*p* < 0.05) suppressed the tartrate-resistant acid phosphatase activity and hydroxyproline level as well as the oxidative and inflammatory stress. Data also observed the more potent anti-osteoporotic effect of the combined extract than the commonly used bisphosphonate drug (alendronate). In conclusion, the administration of TV–BH improved the glucocorticoid-induced bone damage, inflammation, and oxidative stress and as a result, it might be a promising therapeutic option for the OP disorder.

## Abstract 2

Many of the functional foods are designed to decrease the risk of chronic diseases like osteoporosis (OP) which is the most common bone disorder affecting millions of people.

## Graphical abstract

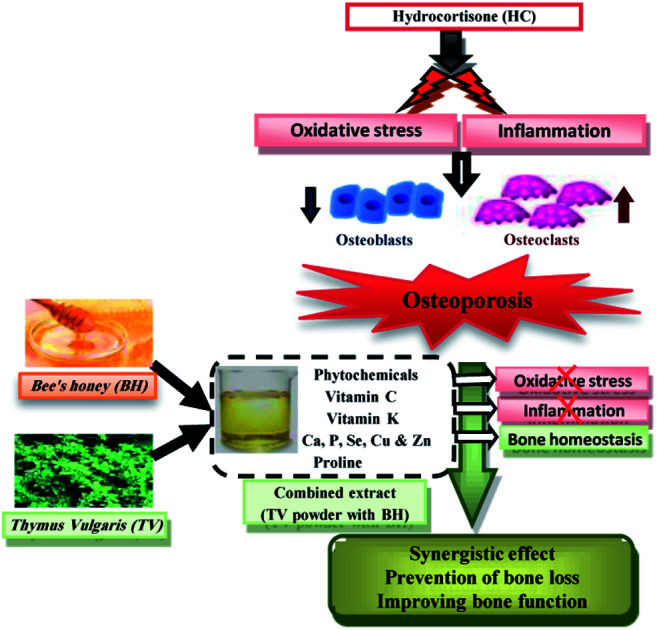

## Introduction

Osteoporosis (OP) is the most prevalent socioeconomic musculoskeletal disease affecting mainly postmenopausal women and older people with significant morbidity and mortality. This disease causes severe pain, disability, and affects females more than males. It is a systematic multifactorial skeletal disorder characterized by deteriorating bone tissues, reduced bone mass, and increased fracture risk. Bone is a supporting framework of the body consisting mainly of the matrix and four cell types, osteoblast, osteoclast, osteocyte and osteoprogenitor.^1^ Osteoblasts synthesize and secrete bone matrix components, mainly collagen, osteocalcin, and alkaline phosphatase (ALP) while osteoclasts are important for bone resorption.^2^ The alteration in the activity or a number of osteoblasts and osteoclasts will disrupt bone homeostasis which is the main cause of OP.^1^ OP is related to many risk factors such as smoking, hormonal factors, diabetes, low calcium and vitamin D intake as well as glucocorticoids.^2^

The long-term treatment with glucocorticoids drugs largely induces OP (secondary OP). These drugs, like hydrocortisone (HC), are most potent anti-inflammatory and immunosuppressive compounds that are widely used in the treatment of various diseases such as autoimmune disorders and rheumatoid arthritis. But they have serious adverse effects on multiple organ systems including bone cells. Because these drugs are able to induce oxidative stress and gene expression of nuclear factor-kappa (NF-κ)B, cyclooxygenase (COX)-2 and tumor necrosis factor (TNF)-α on bone cells. These effects decrease the bone matrix deposition by osteoblasts and increase bone resorption by osteoclasts.^3^ Several medications are used to treat glucocorticoid-induced OP such as calcium and active vitamin D intake, hormone replacement therapy, and bisphosphonates (BPs). The most often treatments are the BPs which are analog for the inorganic pyrophosphate.^4^ Alendronate (ALD), etidronate, clodronate, tiludronate, and pamidronate are the most commonly recommended BP drugs.^5^ These drugs able to attach to the bone surface and inhibit the osteoclasts function by different mechanisms.^4^ However, they are associated with certain side effects such as flu-like symptoms, jaw osteonecrosis, ulcers in the gastrointestinal tract, and they are not safe for patients with severe renal impairment.^4,5^ Therefore, searching for new natural alternative therapies with fewer or no side effects has become important to treat OP.

Thyme (*Thymus vulgaris*, TV) is one of the human nutritional plants belonging to the Lamiaceae family. It was cultivated in many countries such as Iran, Spain, France, Greece, Portugal, and Italy.^6^ Its fresh or dried leaves can be used as a spice and its essential oils used in cosmetics and food additives. TV has ancient traditional uses in the treatment of coughs, headaches, diarrhea, worms, constipation, kidney malfunction, and cancer. In addition, it has various valuable effects, such as antiseptics, antispasmodic, antioxidants, bactericide, and antihelmintic properties. These effects are related to its constituents such as carvacrol, flavonoids, eugenol, thymol, phenols, and luteolin.^7^

Bee's honey (BH) is a natural food known since ancient times by the ancient Egyptians, Greeks, and Chinese and it has been mentioned in the Islamic Hadith (saying of the prophet Mohamed). It has potential uses in medicine due to its anti-microbial, acute and chronic wound healing,^8^ antioxidant, anti-inflammatory and anti-gastritis effects. The flowers used by bees are responsible for the nutritional values and the chemical constituents of BH, which mainly (95–97%) are sugars. In addition, it contains a mixture of different constituents such as proteins, enzymes, vitamins, polyphenols, minerals, organic and amino acids.^9^

The excessive intake of vegetables and fruits has been correlated with a decrease in the risk of various diseases like OP.^10^ Various studies were conducted to study the anti-osteoporotic effects of the polyphenol-rich foods.^11–13^ However, few of them were examined the effect of BH or TV (not the Greek type) on this bone disorder.^14^ Hence there are no previous studies investigating the anti-osteoporotic effect of the combination between TV and BH (TV–BH), the present work evaluated this effect on the HC-induced OP *in vitro*. The *in vitro* antioxidant and anti-inflammatory potentials of TVE were proved recently by the authors^15^ and these two activities were reported for BH.^9^ Therefore, TV–BH may be a promising therapy for OP disease, which is highly associated with the inflammatory and oxidative stress.^3^ In addition, the present study examined certain constituents in TVE and BH useful for bone formation and health to explain the probable anti-osteoporotic role better.

## Materials and methods

### Materials

Folin–ciocalteau reagent, 4-hydroxycinnamic acid (4-HCA), catechin, quercetin (QR), butylated hydroxytoluene (BHT), 2,2-azino-bis(3-ethylbenzthiazoline-6-sulfonic acid (ABTS), 2′,7′-dichlorofluorescin diacetate (DCFH-DA) probe, HC, thiobarbituric acid (TBA), reduced glutathione (GSH), propidium iodide (PI), collagenase type I, 3-(4,5-dimethylthiazol-2yl-)-2,5-diphenyl tetrazolium bromide (MTT), and ALD drug were obtained from Sigma-Aldrich (St. Louis, MO, USA). Roswell Park Memorial Institute (RPMI)-1640 medium, Dulbecco's Modified Eagle Medium (DMEM), α-Minimum Essential Medium Eagle (MEM), trypsin and fetal bovine serum (FBS) were purchased from Lonza, USA. Gene JET RNA purification kit, cDNA synthesis kit, 2X SYBR green master mix kit and protease inhibitor cocktails (PIC) were obtained from ThermoScientific, USA. Osteocalcin ELISA Kit was supplied from Nordic Bioscience Diagnostics (N-MID, Denmark). ALP colorimetric kit was purchased from Biosystems (Barcelona, Spain). The phosphorous colorimetric kit was obtained from Chema Diagnostica (Campania, Italy). TNF-α ELISA kit was purchased from RayBiotech, USA and collagen type IV (COL 4) ELISA kit was supplied from Kamiya Biomedical, US. Primers for NF-κB and COX-2 were purchased from Bioneer, Korea. Other chemicals were obtained with a high grade.

### Extract preparation

The dried TV plant (NCBI:txid49992) was imported from Greece and BH was purchased from a local market in Egypt, then both were authenticated by expert botanist before their use to prepare TVE and TV–BH. The BH produced by *Apis mellifera* bees (NCBI:txid7460) from the citrus fruits nectar.

The TVE was prepared as described previously.^15^ In brief; 50 g of the dried TV plant was powdered, mixed for 10 min with 250 ml of distilled water using an electric blender and soaked for 24 h at 25 °C. Then the solution was filtered and the residues were extracted again. The filtrates were combined, freeze-dried by the lyophilizer (Telstar, Terrassa, Spain), and the powder (TVE, the yield of 9%) was kept in a dark bottle at −20 °C until used.

For preparing the combined extract (TV–BH), 4 g of BH was mixed with 1 g of TV powder and left at 4 °C for 1 week prior to use. The studied extracts were dissolved in distilled water and filtered using syringe filters (0.22 μm) before using in the analyses.

### Extracts characterization

#### Phytochemicals and minerals content

Phytochemicals (phenolics, flavonoids, anthocyanins, and flavonols) and minerals (Cu, Zn, Se, Ca, and P) concentrations in TVE and BH were assessed. Folin–ciocalteau method was used for quantification of the total phenolics using 4-HCA calibration curve and the absorbance was recorded at 750 nm.^16^ Flavonoid content was quantified by mixing 0.2 ml of each extract with sodium nitrite (5%) and AlCl_3_ (10%) and the absorbance was read at 510 nm using catechin as standard.^17^ Anthocyanins were assessed using the pH-differential method that depends on the ability of these pigments to do reversible structural transformations upon the change in the pH.^18^ The sample was dissolved in two buffer solutions at pH 1.0 and pH 4.5 and the absorbance at each pH was measured at 510 and 700 nm. The absorbance of the sample (*A*_s_) was calculated from the equation: [*A*_s_ = (*A*_510_ − *A*_700_)_pH 1.0_ − (*A*_510_ − *A*_700_)_pH 4.5_], whereas the concentration of the anthocyanins pigments was determined as cyanidin-3-glucoside (Cy-3-glc) equivalent from the equation: [anthocyanin pigment = (*A*_s_ × MW × DF × 1000)/(*ε* × sample weight)], where MW and *ε* is the molecular weight and the molar absorptivity of Cy-3-glc, respectively, DF is the sample dilution factor. Total flavonol content was determined using 2% AlCl_3_ and 50 g l^−1^ sodium acetate solutions.^19^ The absorbance was read at 440 nm and the total flavonol concentration was calculated using the QR calibration curve.

For mineral analysis, 1 g of TVE or BH was digested in nitric acid (55%) and perchloric acid (70%) for 1 h at 100 °C. The digested solution was cooled, filtered, and diluted to 10 ml, and then an aliquot of this solution was analyzed using Atomic Absorption Spectrophotometer (Perkin Elmer model 2380, Norwalk, CT, USA).

#### HPLC analysis for identification and quantification of phenolics

Twenty microliters of TVE or BH had separated on 100 mm × 4.6 mm Zorbax Eclipse plusC18 column (Agilent Technologies, Palo Alto, CA, USA). The separation was performed using ternary linear elution gradient with 0.2% H_3_PO_4_, methanol, and acetonitrile at 284 nm. Pure gold standards were run in similar conditions of chromatography to match the retention items.

### Proline and vitamin content

Proline content was determined colorimetrically by the ninhydrin-based method.^20^ It was extracted from TVE or BH using 300 μL of pure ethanol and the solution was heated at 95 °C then mixed with an acid ninhydrin solution (1% ninhydrin, 60% ethanol, 20% acetic acid). The absorbance was recorded at 520 nm after 40 min incubation at 95 °C. The proline content was calculated from the calibration curve of standard proline.

The concentration of vitamin C and K was estimated in TVE or BH by colorimetric methods using 2,4 dinitrophenylhydrazine (2,4 DNPH) and standard vitamins.^21,22^ For vitamin C determination, the deproteinized extract or standard was mixed and incubated with a mixture of 2,4-DNPH (3%), thiourea (0.4%), CuSO_4_ (0.05%) and H_2_SO_4_ (65%) at 37 °C for 1.5 h. At the end of the incubation period, H_2_SO_4_ was added and the absorbance of the colored solution was read at 520 nm after 30 min. Vitamin K was quantified by mixing and incubating 1 ml of each extract or standard with 0.25% 2,4-DNPH and ethanol (70%) at 75 °C for 15 min with vigorous shaking. Then alcoholic ammonia was added and the absorbance of the produced color was recorded after 15 min at 635 nm.

### *In vitro* antioxidant activities

The antioxidant activities of TVE, BH, or TV–BH were evaluated *in vitro* using different assays, including the total antioxidant capacity (TAC), β-carotene-linoleate bleaching, and ABTS^+^ radical cation-decolorization assays.

The TAC of the single and combined extracts was determined using a mixture of 28 mM sodium phosphate, 0.6 M H_2_SO_4_, and 4 mM ammonium molybdate. This mixture was incubated with each of the single or combined extract, standard antioxidant (BHT) or water (blank) at 95 °C for 90 min; then the absorbance was measured at 695 nm.^23^

The β-carotene-linoleate bleaching assay examines the anti-lipid peroxidation activity of the samples using an emulsion from β-carotene, linoleic acid and Tween-80.^24^ The ability of the extract to inhibit the radicals that produced from linoleic acid oxidation as indicated by the decrease in the bleaching rate of β-carotene was measured. The absorbance (*a*, *b*) was read at 490 nm immediately and after 180 min (*t*), respectively. Then the degradation rate (DR) of the extract, standard (BHT), and control (water instead of the extract) was calculated from the equation: [DR = ln(*a*/*b*) × (1/*t*)]. The antioxidant activity was calculated as % of inhibition using the formula: antioxidant activity (%) = (DR_control_ − DR_extract_/DR_control_) × 100. The extract concentration that made inhibition of the β-carotene bleaching by 50% (IC_50_ value) was calculated.

The ABTS^+^ radical cation-decolorization assay assesses the loss of the blue-green color of ABTS^+^ radical upon its reduction by the antioxidants to ABTS.^25^ ABTS^+^ radical was prepared before mixing with the single or combined extract by incubating ABTS (7 mM) with potassium persulphate (140 mM) for 16 h at 25 °C in the dark. The absorbance was read at 734 nm and the % of ABTS^+^ radical inhibition was calculated for IC_50_ value determination and the results were compared with BHT.

### Bone cell isolation and cultivation

The bone cells were isolated from the sterilized femur and tibia of three albino male rats (71–73 g) following the method of Wong *et al.*^26^ with some modifications. All animal procedures were performed in accordance with the Guidelines for Care and Use of Laboratory Animals of Alexandria University and City for Scientific Research and Technology Applications (SRTA-City). All animal procedures were approved by the Animal Ethics Committee of the National Health and Medical Research Council policies and the Ministry of Health and Population, Egypt.

Bones were dissected into 1 mm pieces and washed well to remove blood, marrow cells, and any soft tissues. Bone pieces were placed in DMEM containing 100 U ml^−1^ penicillin/streptomycin for 20 min in a CO_2_ incubator (New Brunswick Scientific, Netherlands) at 37 °C, 5% CO_2_ and 90% relative humidity. Bone cells were successfully isolated by digestion with 100 U ml^−1^ collagenase type I and 25% trypsin. Cells were cultured with α-MEM containing 20% FBS in 6-well culture plate in a CO_2_ incubator at the same previous conditions. After 24 h, the medium was aspirated and replaced daily thereafter until they reached confluence (90%) within 3 weeks. Then trypsinization was done for cells using trypsin (25%) containing EDTA (0.02%). After washing, cells were tested for the viability and counted using 0.5% trypan blue stain.

### Cytotoxicity assay

About 1 × 10^4^ bone cells were seeded per well in 96-well plate and 100 μL of serial dilutions of each single or combined extract or ALD were added and incubated in 5% CO_2_ incubator. After 72 h, 20 μL of MTT (5 mg ml^−1^ in phosphate buffered saline, PBS) was added per well and incubated in a CO_2_ incubator for further 3 h. Then 100 μL of DMSO was added to each pellet (formazan crystals, MTT byproduct) for solubilization and the absorbance was read at 570 nm using an ELISA reader (BMG LabTech, Germany). Cell viability was determined and a relation between it and each of the studied extract or standard drug concentrations was plotted for calculating the safe concentrations (EC_100_, 100% cell viability).

### Development of OP *in vitro* model and treatment protocol

Cells from bone were incubated with serial concentrations of HC (1.25, 2.5, 5, 10, 20 mg ml^−1^). After 48 h, the % of bone cell damage and bone cell turnover (formation and resorption) markers were assessed at each HC concentration. Then the IC_50_ values were calculated for the % cell damage and from the % inhibition or depletion values of each bone turnover marker. The best concentration of HC that was selected for the establishment of the OP *in vitro* model was calculated as the mean value for these IC_50_ values. After induction, cells were treated with the safe concentration (EC_100_) of each single or combined extract or the standard drug. Then cells were morphologically observed using a phase contrast inverted microscope (Olympus, Tokyo, Japan) after 72 h incubation in a CO_2_ incubator. The % of bone cell damage, bone cell turnover markers, inflammatory mediators, and oxidative stress parameters were evaluated as follows.

### Evaluation of bone cell damage by PI-staining assay

PI stain was used to determine the percentage of bone cell damage due to its ability to stain the chromatin of dead cells only. About (1 × 10^4^) of the untreated and HC-, TVE-, BH-, (TV–BH)-, and ALD-treated cells in 96-well plate were washed twice and then suspended in 100 μL PBS containing 10 μg ml^−1^ PI. After 30 min, plates were read using a fluorescence plate reader (BMG LabTech, Germany) at 520 nm emission and 490 nm excitation.

### Biochemical markers of bone cell turnover

The medium of the untreated and HC-, TVE-, BH-, (TV–BH)-, or ALD-treated bone cells were removed and used in the quantification of osteocalcin level. The bone cell lysate was prepared by washing cells twice with PBS containing PIC then cells were treated with trypsin, washed twice, and suspended. Cells were lysed in PBS using a sonicator (2 cycles, each of 10 s duration) then cell lysate was centrifuged at 600*g* for 5 min, suspended in PBS, and used for the biochemical assays.

### Bone cell formation markers

The markers of bone cell formation were assessed, including mineralization, osteocalcin, phosphorous, ALP, and collagen content.

The production of extracellular matrix calcium deposits by osteoblasts (mineralization, bone nodule formation) was detected by alizarin red staining.^27^ Bone cell monolayers in a 6-well plate were fixed in 10% formaldehyde for 15 min at 25 °C and stained with 40 mM alizarin red (pH 4.1). The stained cells were visualized and photographed with the phase contrast microscope. Then the absorbance was read at 405 nm and the concentration was determined using the hydroxyapatite (calcium phosphate tribasic, Ca_10_[PO_4_]_6_[OH]_2_) calibration curve. The osteocalcin concentration was determined by specific ELISA Kit using two highly specific capture and detection monoclonal antibodies following the manual instructions. Phosphorous was assessed in bone cell lysates using the Chema Diagnostica colorimetric kit following the manufacturer's protocol. The method based on the formation of the colorless phosphomolybdate complex in an acidic medium which can be measured at 340 nm. The intracellular ALP was determined in the bone cell extract that was prepared by mixing 100 μL of 0.1% sodium dodecyl sulfate (SDS) with the cell lysates at 4 °C for 40 min. ALP activity was measured colorimetrically using 6 mM 4-nitrophenyl phosphate (4-NPP) as a substrate and the procedure was continued according to the ALP colorimetric kit manual. The increase in absorbance was measured per min for 3 min at 405 nm. One unit of ALP activity is defined as the amount of enzyme which produced 1 μmol of 4-NP/min and the specific activity is expressed as units of enzyme activity per mg of protein. Collagen content was determined in the cell lysates using the specific ELISA kit following the manual protocol.

### Bone cell resorption markers

Tartrate-resistant acid phosphatase (TRAP) activity and hydroxyproline content were determined as markers of the bone cell resorption.

The TRAP activity was determined in the cell extract (prepared as described in the ALP assay) according to Burstone method.^28^ The solution of 4-NPP (2.2 mg ml^−1^) in 0.5 M sodium tartrate solution containing 0.5 M sodium acetate and adjusted to pH 6.1 was used as a substrate. The absorbance was read at 405 nm and the enzyme activity was determined using 4-NP calibration curve. Then the specific activity of the enzyme that was defined as the amount of enzyme required to hydrolyze 1 μmol of 4-NPP per min per mg protein at 37 °C was calculated. Hydroxyproline content in cell lysates was measured using chloramines T and dimethylaminoborane solutions.^29^ The absorbance of the produced color was read spectrophotometrically at 560 nm and the concentration was determined from the hydroxyproline calibration curve.

### Determination of the oxidative stress parameters

The intracellular reactive oxygen species (ROS) level, lipid peroxidation, GSH, and the antioxidant enzymes were investigated in the untreated control, HC-, TVE-, BH-, (TV–BH)-, and ALD-treated bone cells.

The ROS level was assessed using DCFH-DA fluorescent probe method.^30^ This method is extremely sensitive to the change in the cellular redox state. Bone cells were preloaded with 5 μM of DCFH-DA at 37 °C for 30 min. The cellular esterases cleave DCFH-DA producing a H_2_DCF non-fluorescent product, which was oxidized by ROS to DCF fluorescent molecules. The intensity of the produced fluorescence was examined by the flow cytometry (FACS Calibur, Becton Dickinson, USA) at an excitation (488 nm) and an emission (530 nm) wavelength. Results were analyzed using the CELLQuest program (Becton Dickinson).

The medium of 1 × 10^6^ bone cells was used in determining the lipid peroxidation and bone cell lysate was used in the assay of the antioxidant indices and total protein levels. The degree of lipid peroxidation was assessed by determination of the TBA reactive substances (TBARS) level.^31^ GSH level was measured using Ellman's reagent (5,5′-dithiobis2-nitrobenzoic acid) for production of a yellow-colored product with a maximum absorbance at 412 nm.^32^ Glutathione peroxidase (GPX) activity was determined colorimetrically using GSH and cumene hydroperoxide as substrates.^33^ The activity of Cu/Zn superoxide dismutase (SOD) was assessed using the pyrogallol autoxidation method,^34^ in which the change in absorbance during 2 min was measured at 420 nm. The unit of activity is defined as the amount of enzyme that inhibits the rate of autoxidation of 20 mM pyrogallol by 50% under standard conditions.

### Determination of the inflammatory mediators

About 1 × 10^6^ of the untreated bone cells and cells that treated with HC, TVE, BH, (TV–BH), or ALD were centrifuged at 600*g* for 10 min. The culture medium per well was collected and used for quantification of the TNF-α protein following the manufacturer's protocol of the ELISA kit. Also, NO level was determined in the culture medium by the Griess reaction, which produced colored azo dye with a maximum absorbance at 490 nm.^35^ While cells were detached with trypsin–EDTA; then washed twice with PBS and used for total RNA extraction following the manual protocol of Gene JET RNA Purification Kit. The cDNA was synthesized from 1 μg of each RNA sample by reverse transcriptase-PCR using the cDNA Synthesis Kit. Then the NF-κB and COX-2 gene expression were determined by real-time quantitative polymerase chain reaction (qRT-PCR, Qiagen, Germany). Levels of the gene expressions of target genes and reference gene (β-actin) were quantified using the gene-specific forward and reverse primers. The following primers were used: NF-κB, forward 5′-CCTCGGGGTCCTACTCAAGA-3′, reverse: 5′-AGAGGTGTCGTCCCATCGTA-3′; COX-2, forward: 5′-CCCATGTCAAAACCGTGGTG-3′, reverse: 5′-CTTGTCAGGAATCTCGGCGT-3′; and β-actin, forward: 5′-CCACCATGTACCCAGGCATT-3′, reverse: 5′-CCTAGAAGCATTTGCGGTGC-3′.

The reaction mixture enclosed 0.3 μl of each forward and reverse primer (10 μM), 12.5 μL of the 2X SYBR green master mix, and 50 ng cDNA templates. Then, the total volume was completed to 25 μl with nuclease-free water. The qPCR program was applied as one cycle of enzyme activation for 15 min at 95 °C followed by 40 cycles of denaturation for 15 s at 95 °C, annealing for 1 min at 60 °C and extension for 30 s at 72 °C. The expression of target genes was calculated using the comparative Ct method (threshold cycle number at cross-point between amplification plot and threshold). The CT values of each target gene were normalized to that of β-actin according to manufacturer's manual and the change in the gene expression (2^−ΔΔCT^) was calculated.

### Total protein content

The total protein content of the cell lysate and cell extract (SDS-treated cell lysate) was determined using Bradford Coomassie brilliant blue assay.^36^ The produced blue colored complex was read at 595 nm. The concentration of the protein was determined from the bovine serum albumin standard curve.

### Impact evaluation of TV and BH combination

The combination of food extracts may give higher (synergistic), lower (antagonistic), or no new (additive) effect compared to the effect of the single extracts. Evaluation of this new effect can be done through determination of the combination index (CI) value. Here the CompuSyn software was used to calculate the CI values for the *in vitro* antioxidant activities and for other studied parameters, these values were calculated by dividing the predictable value on the practical value. The predictable value between extract 1 and extract 2 is determined as [(practical value for extract 1)/(control value)] × [(practical value for extract 2)/(control value)] × (control value).^15^ The CI value may be equal to 1 (additive effect), <1 (synergistic effect) or >1 (antagonistic effect).^37,38^

### Data analysis

The results are presented as mean ± SE and the comparison between the mean values of different treatments was done using one-way ANOVA *via* Duncan's test. The significance was determined at *p* < 0.05 and the analysis was performed for three measurements using SPSS software version 16. GraphPad PRISM software version 6 with appropriate 95% confidence interval was used for determination of the IC_50_ and EC100 values. The CI value and the CI plots for the *in vitro* antioxidant assays were achieved by the CompuSyn software (ComboSyn, Inc, Paramus, NJ).

## Results and discussion

### Extracts constituents

**Fig. 1:**
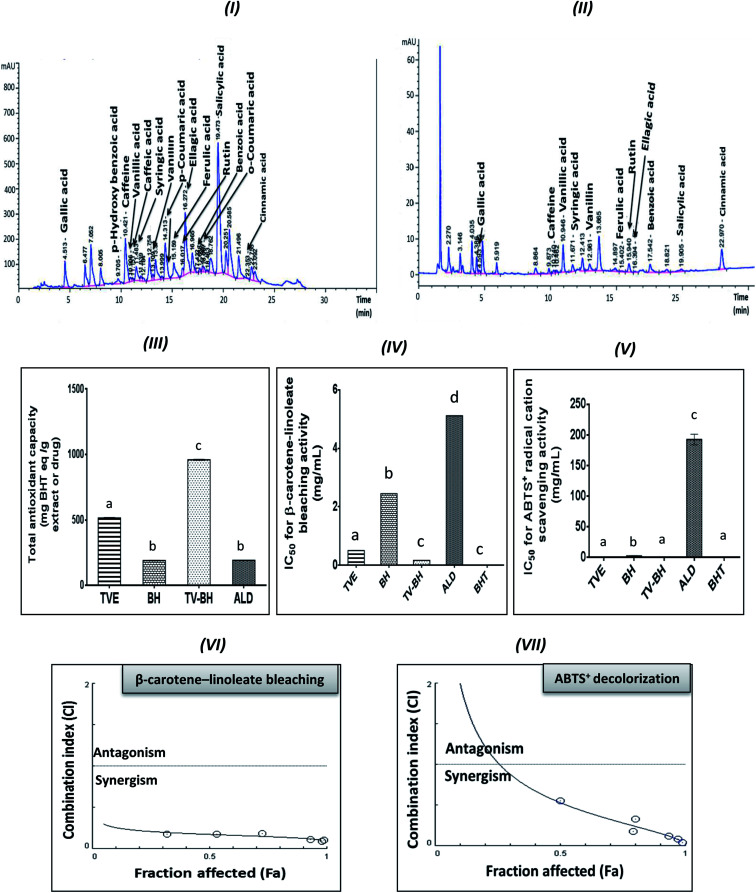
HPLC chromatograms of the phenolic compounds in the Greek *Thymus vulgaris* extract (TVE) and bee's honey (BH) and the *in vitro* antioxidant activities of them and the combined extract (TV–BH) in comparison with the alendronate (ALD) and the butylated hydroxytoluene (BHT). (I and II) HPLC chromatogram of TVE and BH, respectively (III) total antioxidant capacity (IV) β-carotene-linoleate bleaching activity (V) ABTS^+^ (2,2-azino-bis(3-ethylbenzthiazoline-6-sulfonic acid) radical cation scavenging activity (VI and VII) Combination index (CI) plots, Fa is the % of inhibition values of β-carotene and ABTS^+^ radical, respectively at different CI values. Data are presented as mean ± SE (*n* = 3). Different letters for the same parameter are significantly different at *p* < 0.05.

Table 1 represented the chemical constituents of TVE and BH. The results revealed the presence of considerable amounts of proline, phenolics, flavonoids, anthocyanins, flavonols, and important minerals and vitamins in TVE. BH contains these compounds except flavonoids. In addition, the HPLC analysis detected fifteen phenolic compounds in TVE (Fig. 1I) and twelve ones in BH (Fig. 1II) using identified phenolic standards (Table 1) by comparing their particular retention times. These constituents are necessary for bone health and strength.

**Table 1:** Composition of some constituents in the Greek *Thymus vulgaris* water extract (TVE) and bee's honey (BH)^a^

BH	TVE	Constituent
**Amino acids (μmol g** ^**−1**^ **extract)**		
0.812 ± 0.101^b^	1.859 ± 0.159^a^	Proline

**Phytochemicals**		
6.561 ± 0.056^b^	627.194 ± 0.111^a^	Phenolics (mg 4-HCA eq. g^−1^ extract)
ND	135.674 ± 0.000	Flavonoids (mg catechin eq. g^−1^ extract)
ND	1.076 ± 0.165	Anthocyanins (mg Cy-3-glc eq. g^−1^ extract)
ND	41.830 ± 0.000	Flavonols (mg QR eq. g^−1^ extract)

**Phenolics (μg g** ^**−1**^ **extract)**		
11.7 ± 0.009^b^	214.041 ± 0.990^a^	Gallic acid
ND	302.360 ± 1.800	*p*-Hydroxybenzoic acid
10.300 ± 0.543^b^	589.863 ± 0.876^a^	Caffeine
86.200 ± 0.200^b^	45.166 ± 0.166^a^	Vanillic acid
18.400 ± 0.400^b^	11.922 ± 0.800^a^	Caffeic acid
23.300 ± 0.622^b^	147.263 ± 0.655^a^	Syringic acid
23.300 ± 0.511^b^	37.711 ± 2.333^a^	Vanillin
ND	182.656 ± 3.000	*p*-Coumaric acid
2.700 ± 0.004^b^	184.660 ± 0.233^a^	Ferulic acid
90.600 ± 1.003^b^	241.111 ± 2.544^a^	Rutin
4.623 ± 0.788^b^	1646.741 ± 13.456^a^	Ellagic acid
406.005 ± 4.773^b^	37.777 ± 0.444^a^	Benzoic acid
ND	21.768 ± 0.099	*o*-Coumaric acid
36.893 ± 1.223^b^	4179.990 ± 3.655^a^	Salicylic acid
27.643 ± 3.754^b^	1.045 ± 0.001^a^	Cinnamic acid

**Vitamins (mg g** ^**−1**^ **extract)**		
0.711 ± 0.236^b^	3.242 ± 0.361^a^	C
11.005 ± 0.344^b^	146.875 ± 9.375^a^	K

**Minerals (μg g** ^**−1**^ **extract)**		
66.600 ± 0.012^b^	11.940 ± 0.001^a^	Cu
11.600 ± 0.003^b^	284.000 ± 0.000^a^	Zn
2.998 ± 0.010^b^	254.000 ± 0.002^a^	Se
22.000 ± 0.004^b^	3000.000 ± 0.001^a^	Ca
20.000 ± 0.059^b^	7.016 ± 0.877^a^	P

a Results are presented as mean ± SE (*n* = 3). Different letters in the same row are significantly different at *p* < 0.05. BH, bee's honey; Cy-3-glc, cyanidin-3-glucoside; 4-HCA, 4-hydroxycinnamic acid; QR, quercetin; TVE, Greek *Thymus vulgaris* water extract; ND, not detected.

### *In vitro* antioxidant activity

The graphs III–V in Fig. 1 clearly showed the potent antioxidant activities of TVE and BH more than ALD. The figure also demonstrated that the potency of TVE was more than BH. In comparison with the standard antioxidant (BHT), TVE exhibited similar ABTS^+^ radical cation scavenging activity, but BH demonstrated less activity. Regarding the potency of the β-carotene-linoleate bleaching, TVE and BH had activities lower than BHT. These results confirm our previously published data that revealed the anti-radical and anti-lipid peroxidation potentials of TVE.^15^ In addition, the antioxidant activity of Egyptian^39^ and Indian^40^ TV and BH^41^ were previously confirmed.

On the other hand, TV–BH showed the synergistic bleaching ability to the β-carotene-linoleate and synergistic scavenging activity for the ABTS^+^ radical with higher potency compared to ALD. Using Chou–Talalay analysis, the CI graph was plotted (Fig. 1VI and VII) and all the CI values were less than 1 (synergistic effect).

The antioxidant effect of TVE and BH may be related to their constituents, including phenolic compounds and vitamin C (Table 1). The highest content of these compounds in TVE gave it the superior antioxidant activities. Phenolic compounds are the most important and potent antioxidants in plants and other foods that protect from the oxidative stress damage and decrease the risk of chronic diseases. The anthocyanins and flavonols content of TVE are important functional food constituents with potent antioxidant activity.^42^ In addition, the identified phenolic acids in both single extracts (Table 1) were reported previously as potent ROS scavengers.^43^ Also, the presence of vitamin C with the phenolic compounds in the studied extracts can synergistically amplify their antioxidant potential.^42,44^ This action helps in more scavenging and detoxification of the ROS and decreases its level.

The combination of food extracts may help in the elevation of the antioxidant power of the mixture more than the single extracts.^15,45^ This may be related to specific kinds of interactions occurred between the constituents of the single food extracts.^46^ Therefore, the interaction between TV and BH constituents after combination enhanced the antioxidant capability of the combined extract (TV–BH). However, the exact interaction to which this effect owed to need further investigations.

### Bone cell viability and cytotoxic effect of the studied single and combined extracts

The results showed that TVE, BH, and TV–BH had the same safety effect on the bone cells and they were safer than ALD. The values of the EC_100_ were 4.656 ± 0.141, 4.508 ± 0.277, 5.059 ± 0.066, and 1.007 ± 0.007 mg ml^−1^, respectively. The values of IC_50_ as obtained from the MTT assay were 1.500 ± 0.026, 0.749 ± 0.003, 1.459 ± 0.003, and 0.026 ± 0.018 mg ml^−1^, respectively. These values have significantly (*p* < 0.05) differed from each other and mean that the studied single and combined extracts are less cytotoxic than ALD on the bone cells. These properties increase the nutritional value of the studied extracts and gave them great public health importance.

### Improvement of the HC-induced bone turnover disturbance by the studied single and combined extracts

**Fig. 2:**
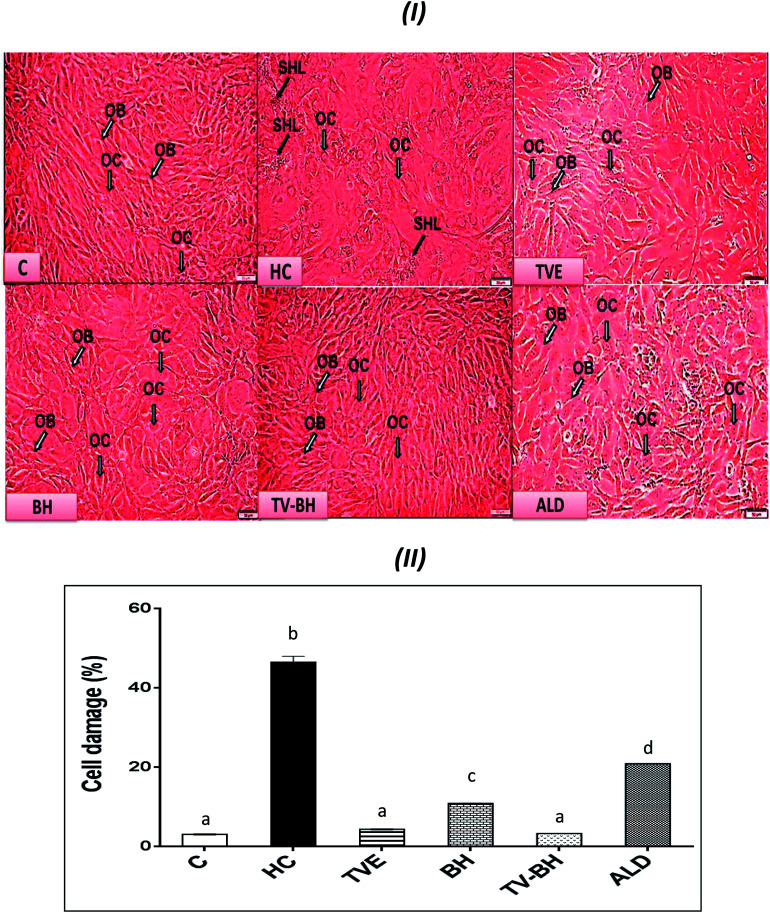
The effect of *Thymus vulgaris* water extracts (TVE), bee's honey (BH), combined extract (TV–BH), and alendronate (ALD) on the hydrocortisone (HC)-induced bone cell damage. (I) Morphological changes in the bone cells as observed under the inverted microscope (II) % of bone cell damage using propidium iodide. C; the untreated control cells, OB; osteoblasts, OC; osteoclasts, SHL; saucer-shaped Howship's lacunae. Data are presented as mean ± SE (*n* = 3). Different letters for the same parameter are significantly different at *p* < 0.05.

**Fig. 3:**
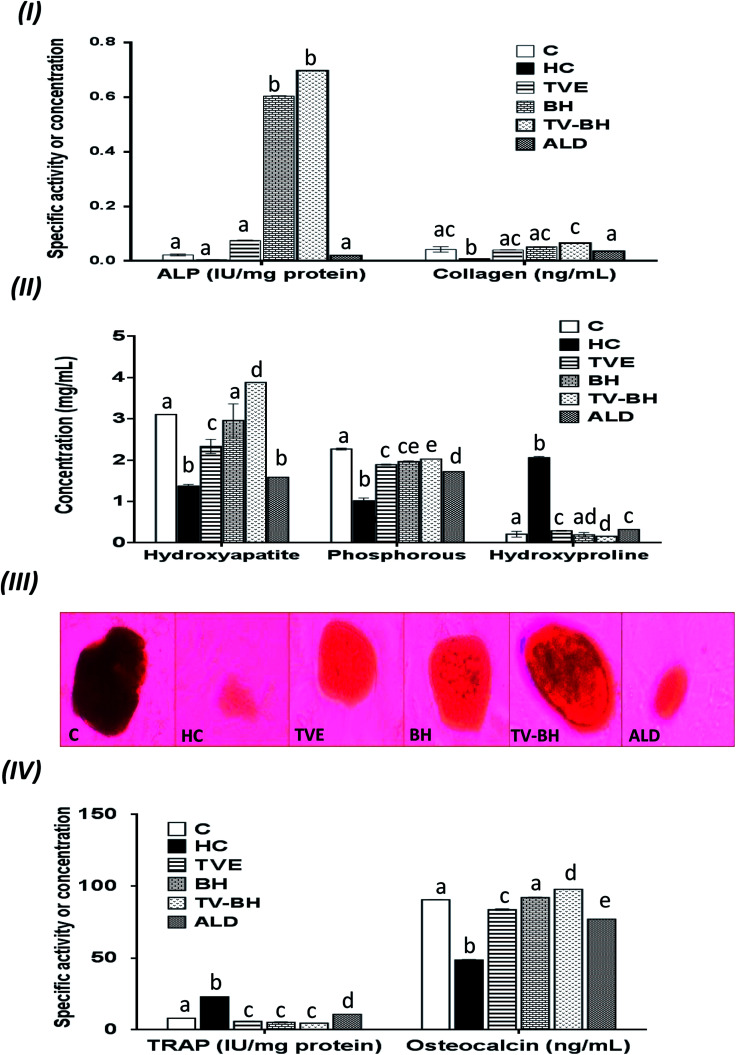
The changes in the bone turnover markers following the treatment with *Thymus vulgaris* extract (TVE), bee's honey (BH), combined extract (TV–BH), and alendronate (ALD) in the hydrocortisone (HC)-induced osteoporosis in the bone cells. (I) alkaline phosphatase (ALP) activity and collagen level (II) hydroxyapatite, phosphorous, and hydroxyproline levels (III) alizarin red S staining of bone nodules. The dark red staining reveals the hydroxyapatite formation (mineralization) (IV) tartrate-resistant acid phosphatase (TRAP) activity and osteocalcin level. C; the untreated control cells. Data are presented as mean ± SE (*n* = 3). Different letters for the same parameter are significantly different at *p* < 0.05.

In the current study, HC was able to induce OP in the bone cells at the concentration of 9.167 ± 2.123 mg ml^−1^ by disrupting the bone homeostasis. This concentration represents the average value of the IC_50_ values for all the studied bone markers at different HC concentrations (Table 2). The results revealed that the order of the bone markers inhibition or depletion by HC was the collagen > osteocalcin > ALP and hydroxyproline > P, hydroxyapatite, and TRAP. Fig. 2 showed the degree of the bone cell damage after their exposure to the HC and the studied treatments. The morphology of the bone cells (Fig. 2I) after HC treatment showed a sharp decrease in the osteoblasts and increase in the osteoclasts. In addition, the degree of the bone cell damage as indicated by PI staining (Fig. 2II) was highly significantly (*p* < 0.05) elevated over the untreated control cells. Fig. 3 showed that HC significantly (*p* < 0.05) down-regulated all the tested bone formation markers and up-regulated all the tested bone resorption markers. These results indicated that the exposure of the bone cells to HC induced disturbance in the bone homeostasis and increased cell damage. This means that HC at the concentration of about 9 mg ml^−1^ able to induce OP in the bone cells. The results are in line with the previous study of Xie *et al.*^47^

**Table 2:** The IC_50_ values for the bone cell damage and the bone turnover markers after the treatment with different concentrations of hydrocortisone^a^

Hydroxyproline	TRAP	Collagen	ALP	P	Osteocalcin	Hydroxyapatite	Cell damage	Bone marker
9.332 ± 0.017^c^	17.175 ± 0.190^d^	1.176 ± 0.153^a^	7.117 ± 1.081^c^	14.606 ± 0.265^d^	4.040 ± 0.299^b^	15.562 ± 0.612^d^	4.330 ± 1.528	IC_50_ (mg ml^−1^)

a Results are expressed as mean ± SE (*n* = 3). Different letters for the bone markers are significantly different at *p* < 0.05. ALP, alkaline phosphatase; P, phosphorous; TRAP, tartrate-resistant acid phosphatase; IC_50_, hydrocortisone concentration that caused 50% cell damage or (inhibition or depletion) for the bone marker.

The treatment of these damaged cells with TVE or TV–BH relieved the bone cells damage through decrease the osteoclasts and increase the osteoblasts numbers (Fig. 2I and II). Results also showed that BH had less improving effect for the bone cell damage. However, the healing effects of all the studied extracts were significantly (*p* < 0.05) superior to ALD. Also, the treatment with either extract significantly (*p* < 0.05) able to increase the bone formation markers and decrease the bone resorption markers as compared to the HC-treated cells (Fig. 3). The enhancing potency of both extracts for the bone turnover parameters was more than ALD. On the other hand, the treatment with TV–BH extract exhibited a synergistic improving effect for the bone turnover parameters (CI < 1, Table 3) except for ALP (CI > 1, antagonistic effect). These results agree with the previous study that was conducted on the combined extract from Egyptian TV with other herbs.^14^

**Table 3:** Combination index values of anti-osteoporotic activities of the combined extract

Effect	(CI)^a^	Parameters
**Bone turnover markers**		
Synergistic	0.572	Hydroxyapatite (mg ml^−1^)
Synergistic	0.870	Osteocalcin (ng ml^−1^)
Synergistic	0.807	Phosphorous (mg ml^−1^)
Antagonistic	2.931	ALP activity (IU mg^−1^ protein)
Synergistic	0.767	Collagen (ng ml^−1^)
Synergistic	0.817	TRAP activity (IU mg^−1^ protein)
Synergistic	0.922	Hydroxyproline (mg ml^−1^)

**Inflammatory mediators**		
Synergistic	0.114	NO (nmol ml^−1^)
Synergistic	0.000	COX-2 (expression fold)
Synergistic	0.000	NF-κB (expression fold)
Synergistic	0.313	TNF-α (ng ml^−1^)

**Oxidative stress parameters**		
Synergistic	0.278	Lipid peroxidation (nmoL ml^−1^)
Synergistic	0.421	GSH (nmoL ml^−1^)
Synergistic	0.945	SOD (IU mg^−1^ protein)
Synergistic	0.381	GPX (IU mg^−1^ protein)

a CI (combination index) value of <1 means synergistic effect; >1 means antagonistic effect; = 1 means additive effect. ALP; alkaline phosphatase, TRAP; tartrate-resistant acid phosphatase, NO; nitric oxide, NF-κB; nuclear factor kappa B, TNF- α; tumor necrosis factor-α, SOD; superoxide dismutase, GPX; glutathione peroxidase.

Excess glucocorticoids induce an imbalance between the osteoblasts and osteoclasts by decrease osteoblasts proliferation and lifespan and increase the osteoclasts lifespan and formation. This will increase the bone resorption and suppress the bone matrix formation. Therefore, the synthesis of osteocalcin, ALP, and collagen (Fig. 3I and IV) by osteoblasts was decreased after exposure the bone cells to the HC. This may cause depletion of the hydroxyapatite and phosphorus content (Fig. 3II and III) due to the importance of these proteins in bone mineralization. Since ALP is an essential enzyme promotes the initial phase of bone mineralization by decreasing the pyrophosphate concentration and increasing the inorganic phosphate concentration. However, osteocalcin can bind strongly to the hydroxyapatite and deposited it in a matrix using collagen fibrils as a template.^2^ In addition, the elevation in the osteoclasts numbers caused secretion of the high amount of TRAP. This enzyme is elevated in OP and helps in the generation of ROS to destroy collagen and other bone matrix proteins. Also, it promotes the attachment of the integrin receptors on the membranes of osteoclasts with the bone matrix for digestion and resulted in the formation of the saucer-shaped Howship's lacunae “SHL” (Fig. 2I). Digestion of bone matrix collagen protein leads to the depletion of its content in bone cells and elevation of the hydroxyproline level.^2,48^

The presence of many anti-osteoporotic agents in TVE and BH such as vitamin C and K, proline, minerals, and phytochemicals (Table 1) enable them to improve the bone damage that induced by HC. Vitamin C plays vital roles in collagen synthesis, osteoblasts regeneration, and inhibits osteoclasts formation.^28^ As a result, the synthesis of the bone extracellular matrix components by osteoblasts such as ALP, collagen, and osteocalcin was increased in TVE- and BH-treated cells. While the digestion of collagen and other bone matrix proteins, hydroxyproline level, and the synthesis of TRAP by osteoclasts were decreased. This means that the treatment with either TVE or BH returns the bone cells homeostasis. Also, the presence of considerable amounts of proline in TVE and BH helps in the synthesis of new collagen protein and improved its content in bone cells. The Ca and P content in TVE and BH (Table 1) is necessary to the hydroxyapatite crystals formation and bone calcification.^7^ Therefore, the treatment of the damaged bone cells with these extracts led to the elevation of P and hydroxyapatite levels as compared with the HC-treated cells. These findings are in accordance with the previous studies that were examined the Egyptian TV^49^ and BH.^50^ Zn is another essential element in the studied extracts; its anti-osteoporotic effect can be supported by an elevation in the bone mineralization and reduce the osteoclast resorption activities.^51^ TVE and BH also contain vitamin K which is a crucial vitamin for bone mineralization^2^ and synthesis of bone matrix proteins.^28^ Furthermore, the presence of anthocyanins, flavonols, rutin, and phenolic acids in the studied extracts (Table 1) enable them to protect the bone cells from the HC-induced bone resorption.^10–13,52^

Regarding the combination between TVE and BH, such combination maximized (synergistic action) the increase in the bone cell formation and the reduction in the bone cell resorption markers (Table 3). This may be owed to the interaction between the different constituents in both extracts including phenolic compounds, minerals, vitamins, and others.

### Reduction of the HC-induced oxidative stress in bone cells by the studied single and combined extracts

**Fig. 4:**
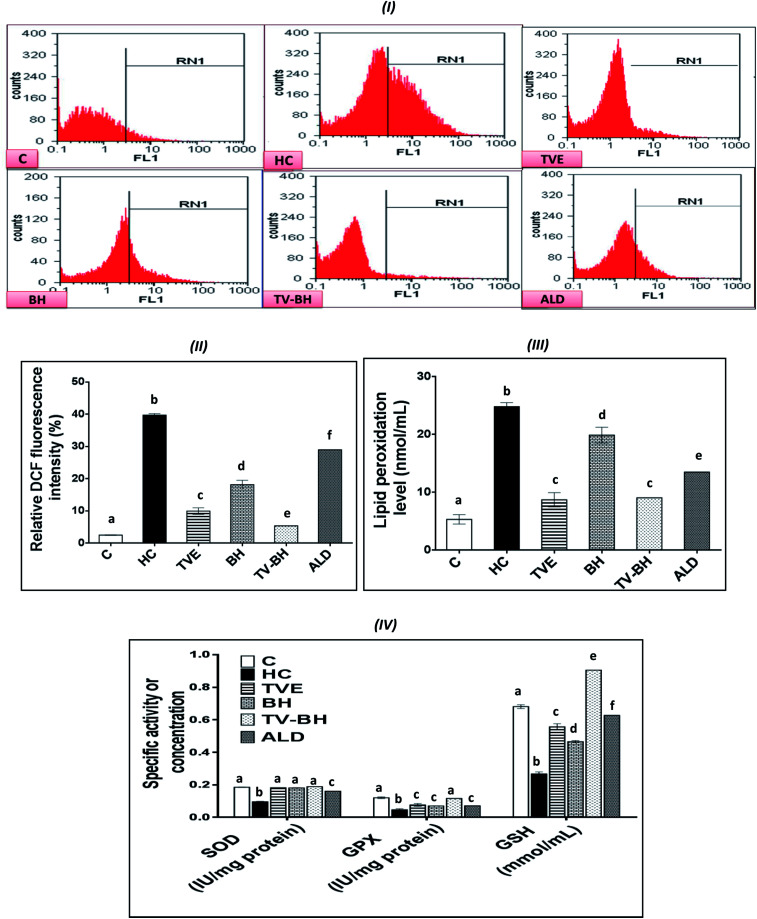
The effect of *Thymus vulgaris* extract (TVE), bee's honey (BH), combined extract (TV–BH), and alendronate (ALD) on the hydrocortisone (HC)-induced oxidative stress in the bone cells. (I) Flow cytometric histograms of the dichlorofluorescein (DCF)-derived fluorescence (II) quantitative results of the flow cytometry (III) lipid peroxidation level (IV) superoxide dismutase (SOD) and glutathione peroxidase (GPX) activities and reduced glutathione (GSH) level. C; the untreated control cells. Data are presented as mean ± SE (*n* = 3). Different letters for the same parameter are significantly different at *p* < 0.05.

The graphs in Fig. 4 showed that HC induced significant (*p* < 0.05) overproduction of ROS (Fig. 4I and II) and lipid peroxidation (Fig. 4III) as compared to the control. Also, it induced a significant (*p* < 0.05) reduction in the level of GSH and the activities of both SOD and GPX (Fig. 4IV). These results indicated that HC induced oxidative stress in bone cells. The balance between ROS and the redox system in bone cells was efficiently preserved after the treatment with TVE and BH. This occurred through significant (*p* < 0.05) depletion in the intracellular ROS and lipid peroxidation levels and significant (*p* < 0.05) elevation in the antioxidant enzymes activities. The data revealed the higher antioxidant potency of TVE than BH while both extracts showed higher or similar antioxidant power as compared to the ALD. These results confirmed our previous data which elucidated the ability of TVE to prevent the LPS-induced oxidative stress in the white blood cells.^15^ Also, the ability of BH to suppress the oxidative stress *in vivo* was reported previously by Halawa *et al.*^53^ Moreover, the present study demonstrated the synergistic (CI < 1) combination of TVE and BH in the reduction of the oxidative stress in bone cells (Table 3). Hence, TV–BH reduced the ROS and lipid peroxidation levels that seemed much narrower than the untreated control group and normalized SOD and GPX activities with potency significantly (*p* < 0.05) greater than ALD.

**Fig. 5:**
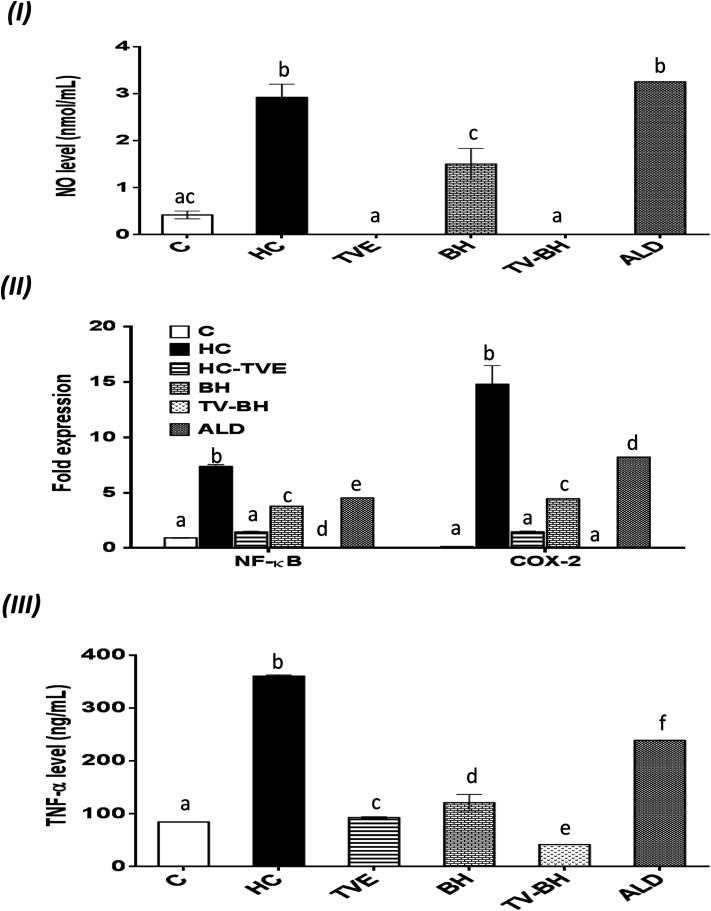
The effect of *Thymus vulgaris* extract (TVE), bee's honey (BH), combined extract (TV–BH), and alendronate (ALD) in the hydrocortisone (HC)-induced inflammation in the bone cells. (I) Nitric oxide (NO) level (II) the gene expression of nuclear factor kappa (NF-κ)B and cyclooxygenase (COX)-2 (III) the protein level of tumor necrosis factor (TNF)-α. C; the untreated control cells. Data are presented as mean ± SE (*n* = 3). Different letters for the same parameter are significantly different at *p* < 0.05.

Glucocorticoids were reported to induce oxidative stress in collagen-producing tissues, such as skin, bone, and tendons in a time and dose-dependent manner and this will involve in their toxicity. This damage occurred by increasing the mitochondrial oxidation and production of the high amount of superoxide radicals. The flow cytometric analysis of the intracellular ROS in bone cells confirms the excessive ROS production after HC treatment (Fig. 4I and II) which produced extreme cellular damage. When the ROS level overwhelms the cellular scavenging ability, cells may undergo necrosis or apoptosis to severe the mitochondrial damage as observed in Fig. 2II. Overproduction of ROS may also result in consumption and depletion of the GSH level in the bone cells.^44^ In addition, SOD, the first line of defense in the cellular antioxidant defense system,^34^ can be inhibited and exhausted by ROS (Fig. 4IV). Further reduction in the SOD activity can be happened due to the overproduction of the hydrogen peroxide as a result of the HC-induced inhibition of the GPX biosynthesis.^54^ GPX activity can also be decreased due to the GSH depletion (Fig. 4IV) and the elevation of NO production (Fig. 5I) in bone cells. The decrease in SOD, GPX, and GSH levels with ROS overproduction in HC-treated cells will affect the cellular biomolecules especially lipids causing lipid peroxidation (Fig. 4III). This will induce disturbance and alteration in the biomembranes fluidity, permeability, and integrity, leading finally to their functional loss.^44^ Oxidative stress plays a role in the initiation and progression of OP by inducing the imbalance between the osteoblastic and osteoclastic activities.^3^

The treatment with TVE and BH significantly (*p* < 0.05) decreased the intracellular ROS level, which revealed the potent scavenging ability of these extracts and confirms their *in vitro* TAC and antiradical activities (Fig. 1III–V). This led to increase in the redox state of the cell and protect the cellular biomolecules from the ROS damage effects. As a result, the lipid peroxidation level was reduced as well as the antioxidant enzymes and GSH were preserved in the TVE- and BH-treated bone cells. TVE showed higher antioxidant potency than BH which might be owed to its ingredients. These healing effects of the studied extracts may be owed to their constituents such as phenolics^44^ and minerals (Table 1).^55,56^ The prevention of the oxidative stress and enhancing the antioxidant defense system in bone cells may lead to inhibition of the bone resorption. Thus, the anti-osteoporotic effect of the studied extracts and their abilities to suppress the HC-induced excessive bone damage could be owed to their antioxidant activities.

The synergistic activity of the TV–BH may be owed to its synergistic TAC and antiradical activities (Fig. 1III–V). This effect probably related to the interaction or the co-existence of the single extracts ingredients and was in line with our recently published results.^15^

### Suppression of the HC-induced inflammation by the studied single and combined extracts

The oxidative stress and the overproduction of ROS in bone cells will signify the bone damage by activation of the inflammatory genes in the cells. The results in Fig. 5 showed that HC induced significant (*p* < 0.05) up-regulation of the NF-κB and COX-2 gene expression and the NO and TNF-α protein levels. The treatment with TVE or BH suppressed the production of these inflammatory mediators. Hence, BH significantly (*p* < 0.05) depleted the level of NO, and the expression of NF-κB, and COX-2 while TVE normalized them (Fig. 5I and II). Also, TNF-α protein level was significantly (*p* < 0.05) decreased after the treatment with these tested extracts (Fig. 5III). However, ALD significantly (*p* < 0.05) suppressed these mediators except the NO level with potency less than TVE or BH. Regarding the combined extract (TV–BH), it exerted synergistic anti-inflammatory activities of all the studied inflammatory mediators (CI < 1, Table 3). The potency of this combined extract was significantly (*p* < 0.05) more than ALD.

The improved level of ROS due to the oxidative stress state that provided in the bone cells by HC may be the reason for the induction of NF-κB gene expression. This pathway amplifies the inflammatory response through enhancing the gene expression of other inflammatory mediators such as iNOS, COX-2, and TNF-α. These inflammatory molecules stimulate more oxidative stress within the cell.^57^ The secretion of inflammatory mediators by bone cells and induction of inflammation are common in OP disease. These mediators act as another cause for bone resorption through activation of the osteoclastogenesis and decrease bone formation.^2^

The potent antioxidant effect of the TVE or BH helps in the reduction of the inflammatory mediators in the bone cells. This may be attributed to their pivotal roles in the scavenging and reduction of the ROS (Fig. 4I and II), the major inducer of the NF-κB pathway. In addition, the phenolic^52^ and minerals^56^ content (Table 1) of these tested extracts may enhance their anti-inflammatory activities. The ability of TVE and BH to control the NF-κB pathway and its related inflammatory mediators gives them a powerful therapeutic potential for the OP and other chronic inflammatory diseases. The superior anti-inflammatory role of the TVE to the BH may be related to its higher antioxidant activities (Fig. 1III–V). In agreement with these findings, our recently published data demonstrated the ability of TVE to decrease the LPS-induced inflammation in the white blood cells.^15^ Moreover, the anti-inflammatory effect of the Indian TV^40^ and BH^41^ was studied previously.

Although this highly anti-inflammatory efficiency of the studied single extracts, the combination of them maximized their actions (synergistic effect). This may be owed to the effect of their components while the exact mechanisms that involved in this synergism need for further investigations.

## Conclusion

This work elucidates that TVE more than BH had an anti-osteoporotic effect in the HC-induced bone damage. The combination of both extracts (TV–BH) maximizes this role and therefore it represents a promising therapeutic alternative to avoid the glucocorticoid-induced OP. This potential effect seems to be mediated by the potent antioxidant and anti-inflammatory activities as well as the direct effect of this combined extract on the bone cell homeostasis. In addition, TV–BH can be used instead of BP drugs due to its safe and most effective role in the bone cells.

## Conflicts of interest

Authors have no conflicts of interest to declare.

## Funding

This research received no specific grant from funding agencies in the public, commercial, or not-for-profit sectors.

## Abbreviations

ABTS: 2,2-Azino-bis(3-ethylbenzthiazoline-6-sulfonic acid
ALD: Alendronate
ALP: Alkaline phosphatase
BH: Bee's honey
BHT: Butylated hydroxytoluene
BPs: Bisphosphonates
CI: Combination index
COL 4: Collagen type IV
COX-2: Cyclooxygenase-2
Cy-3-glc: Cyanidin-3-glucoside
DCFH-DA: 2,7-Dichlorofluorescin diacetate
DMEM: Dulbecco's modified Eagle medium
2,4 DNPH: 2,4 Dinitrophenylhydrazine
DR: Degradation rate
FBS: Fetal bovine serum
GPX: Glutathione peroxidase
GSH: Reduced glutathione
HC: Hydrocortisone
4-HCA: 4-Hydroxycinnamic acid
MEM: α-Minimum essential medium Eagle
MTT: 3-(4,5-Dimethylthiazol-2yl-)-2,5-diphenyl tetrazolium bromide
NF-κB: Nuclear factor-kappa B
4-NPP: 4-Nitrophenyl phosphate
OP: Osteoporosis
PI: Propidium iodide
PIC: Protease inhibitor cocktail
QR: Quercetin
ROS: Reactive oxygen species
RPMI-1640: Roswell Park Memorial Institute
SDS: Sodium dodecyl sulfate
SHL: Saucer-shaped Howship's lacunae
SOD: Superoxide dismutase
TBA: Thiobarbituric acid
TBARS: TBA reactive substances
TNF-α: Tumor necrosis factor
TRAP: Tartrate-resistant acid phosphatase
TV: *Thymus vulgaris*

## Supplementary Material
